# Creative trespasses: intertwining the fashion world and outsider art

**DOI:** 10.1017/S2045796022000749

**Published:** 2023-02-21

**Authors:** Giulia Pettinari

**Affiliations:** Associazione Culturale McZee, Visual art, Macerata, Italy

Throughout the 20th century, mutual influences and fascinations between the world of fashion and artistic languages were frequent and stemmed from different practices: on one hand fashion designers who openly quoted or were inspired by already known works of art, on the other artists who made forays into the world of tailoring by collaborating directly with fashion designers.

The creations made in Italy from 1914 onwards by certain futurist artists who theorised a specific futurist garment (Futurist artist Giacomo Balla studied clothes and fabrics in depth and in September 1914 he published *Il vestito antineutrale* the first Futurist fashion manifesto, which was followed by *Il Manifesto della moda femminile futurista* (1920, signed by Volt) the *Manifesto futurista sulla cravatta italiana* (1933, signed by Scurto and Di Bosso) and the *Manifesto futurista sul cappello italiano* (1933, signed by Marinetti, Monarchi, Prampolini and Somenzi).) come to mind, or of the association that arose, in the surrealist sphere, between the stylist Elsa Schiapparelli, ‘the first avant-garde fashion designer’ (G. Calò, D. Scudero, *Moda e arte. Dal decadentismo al postmoderno*, Gangemi Editore Roma 2009, p. 75.), and artists such as Alberto Giacometti, Man Ray, Louis Aragon and especially Salvador Dalì.

Fruitful collaborations between artists and stylists can also be found in the 1950s and above all in the 1960s: in the French area, it is above all the stylist Yves Saint Laurent who, starting in 1965 with his famous Mondrian Dress, began to draw inspiration from works of art, creating collections conceived as true tributes to contemporary art movements (Pop art look 1966) or to great masters (Picasso, autumn/winter 1979; Matisse, autumn/winter 1981–1984 and Van Gogh, spring/summer 1988).

In these years, fashion became a means of mass communication, and in Italy leading names of the history of fashion such as Missoni, Versace, Ferré, Armani, Valentino emerged, personalities who were very sensitive to the suggestions coming from the history of art, and they themselves protagonists of great exhibitions hosted in the most famous museums in the world. (For example *Valentino a Roma. 45 years of style* (Museo dell'Ara Pacis, Roma, 2007). *Gianni Versace* (Metropolitan Museum of Art di New York, 1997). *Giorgio Armani* (Guggenheim, New York, 2000).)

This aspect is significant in order to reflect on the institutional art–fashion relationship that seems to be increasingly consolidated, also through the sharing of exhibition spaces. Today, established fashion designers exhibit their clothes in major museums, not only in temporary exhibitions (The first was the exhibition dedicated to the career of Yves Saint Laurent held in 1983 at the Metropolitan Museum of Modern Art in New York.) but also within itineraries dedicated to permanent collections; and luxury brands, in turn, set up foundations to promote contemporary art.

The Trussardi Foundation, the Prada Foundation, the Cartier Foundation and the Alda Fendi Foundation are just a few of them. The latter has recently inaugurated its new headquarters, with a renovation of a 17th century palace designed by architect Jean Nouvel. Palazzo Rhinocerhos (web site https://rhinocerosroma.com/galleria/), located in the millenary heart of Rome, between the Palatine and the Bocca della Verità, hosts already important exhibitions.

For some time now, some fashion houses have also revealed an interest in outsider art, which is increasingly participating in those same exhibition and market dynamics that involve contemporary art. In 2015, on the occasion of the publication of a volume dedicated to outsider art edited by the scholar Sara Ugolini (Sara Ugolini (a cura di), *La via più breve non è quella retta. Percorsi nell'outsider art*, L'Harmattan Italia, Torino 2015.), I wrote a contribution focused on this topic, which was followed by a speech during the international conference Into the wild organised by the visual arts department of the University of Bologna. (International Conference *Into the wild. Percorsi nell'arte outsider e contemporanea*, saturday 18 ottobre 2018, MAMbo, Museo d'arte moderna di Bologna. Curated by Stefano Ferrari, Cristina Principale, Carole Tansella and Sara Ugolini.)

Since then, observing the exhibition and market processes, we can say that the boundaries between outsider art and contemporary art have increasingly dissolved. Thus, analysing the working methods of some fashion designers, reading their interviews in trade magazines, we see different approaches and intentions and, consequently, different solutions and working methods. Some fashion designers were inspired by a particular outsider's work, concentrating on formal qualities, while others tried to reproduce atmospheres, trying to convey certain emotions to the audience during the fashion shows. Still others have developed a direct contact with creative workshops or specialised museums, producing special collections born of the collaboration between designers and outsider artists working in an atelier context. In 2009, for instance, Marc Jacobs launched a charity operation in support of the Creative Growth Art Center in Oakland, California, by involving the artists Louis Estape, Dan Miller, William Scott and Gerone Spruill, in the creation of a special limited edition collection of the Marc by Marc Jacobs line available in the brand's flagship shops in the US and Europe. The drawings and paintings created by the four artists have been printed on t-shirts, cotton shopping bags and leather clutches, with all proceeds from the sale going to fund workshop activities. A similar operation was carried out in the British area by James Brett, who collected works by spontaneous artists over a period of 10 years, travelled all over the world, and in 2009 set up the Museum of Everything in London. In the Museum are also included the four artists of the Creative Growth Art Centre mentioned above. On the Museum of Everything's online shop it's possible to purchase a Scott Bag, an Estape Bag, as well as designer clothes whose fabrics reproduce outsider works of art. These are pieces from the collection created by the Clements–Ribeiro duo and inspired by the works of six artists from the Museum of Everything. Iniacio Ribeiro told Vogue in 2011:

‘A mutual friend introduced us to James Brett, the founder of the project, and we were immediately seduced by the idea of collaborating. There's a natural synergy between the art in the Museum and what we try to convey in our collections. Suzanne and I visited the Museum's exhibition in Primrose Hill last year and absolutely loved it. We've been fans of outsider art and curiosities for years.’ (Ella Alexander, *Art Attack*, in «Vogue British», sezione News, 24 agosto 2011. https://www.vogue.co.uk/gallery/clements-ribeiro-museum-of-everything-collaboration)

After researching the museum's archive, the selection fell on six designers of various nationalities: Jean-Jacques Oost (La S Grand Atelier, Belgium), Harald Stoffers and Thomas Beisgen (Atelier der Villa, Germany), Tom Wagener (Cooperations, Luxembourg) and finally Erin Punzel and Ramon Avalos (Creative Growth Art Center, USA). The motifs of their works, printed on pajama-style silk tops, skirts, dresses and trousers, gave rise to the Project 6 autumn/winter 2011 collection, which was presented in London at the Selfridges shop in Oxford Street on the occasion of Exhibition #4.

The works, selected for their articulated composition but also for the diversity of motifs they present, perfectly recall the Clements–Ribeiro style, rich in chromatic and formal contrasts. Thus, the uniqueness and originality of the garments can be seen in the evident union of two styles, that of the respective artist, whose graphic motifs are left intact in the reproduced work, and that of the fashion designer.

Can we assume, then, that the fashion world looks to outsider art in search of new, original and inspiring motifs? This question seems to be confirmed by the words of Japanese fashion designer Rei Kawakubo, founder of the fashion house Comme Des Garçons. In 2014, Kawakubo contacted Raw Vision magazine for some graphic design and communication projects, but already the year before, she collaborated with an outsider artist to create clothes that were later included in the Comme Des Garçons women's autumn/winter 2013 collection. The artist in question is Dan Michiels (1956), who works at the Creativity Explored Studio in San Francisco and was discovered in the pages of the magazine.

‘I feel my own limits after continuing for more then 40 years the act of always looking for something new. […] I think I want the power one can feel when discovering something that is beyond what is seen or felt normally […] This is why I think the artworks created by outsider artists are so fantastic and I deeply respect them. For me, I believe outsider art may be the future.’ (E. M. Gomez, *Raw Ispiration* in «Raw Vision», 83 autumn/fall 2014, p. 4)

The vibrant, psychedelic colours and the intricate geometric patterns developed in two dimensions in Michiels' works fascinated Kawakubo so much that she decided to recreate this dense weave in three dimensions: the Japanese designer printed Michiels' two works on fabric, leaving the original design motifs untouched and working on the volumetric, almost sculptural rendering of the dress, thus creating an ‘unconventional collection that unleashes creativity’ as observed by trade magazines. Fascination and inspiration must be mentioned when analysing Turkish designer Bora Aksu's autumn/winter 2012 collection and Antonio Marras' spring/summer 2013 collection. Both draw inspiration from Henry Darger's In the Realms of the Unreal. Aksu's romantic Vivian Girls walked the catwalk wearing pastel-coloured dresses predominantly in grey, powder pink and beige, which alternated with garments featuring a sudden presence of orange either on the skirt or on the shoulder cover. Embroidery, lace and decorations abounded to evoke a fantastic, childlike world; thus Aksu, borrowing Darger's pastel shades, introduced a strong contrast in the image of his Vivian Girls through the use of heavy, opaque fabrics alternating with light, flowing fabrics in the same garment. Accessories and hairstyles also contributed to the same end (Figs 1–4).

**Fig. 1.**
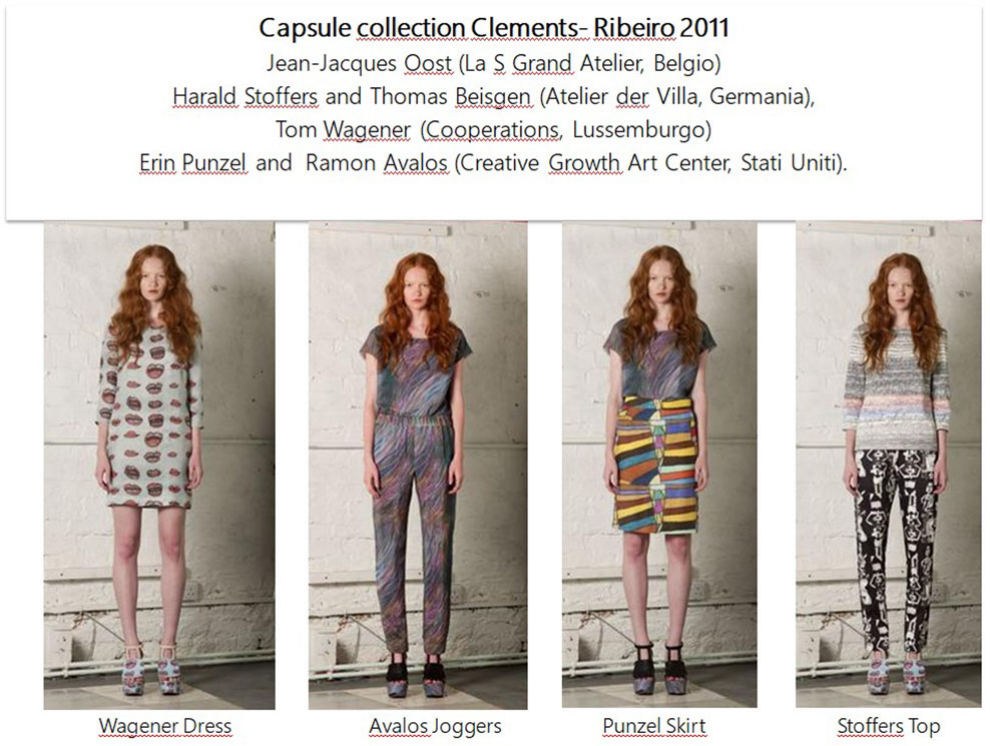
Some dress of capsule collection Clements Ribeiro 2011. Image 1 from: https://shop.musevery.com/collections/clothing?page=4. © 2022 Everything Ltd /The Museum of Everything.

**Fig. 2.**
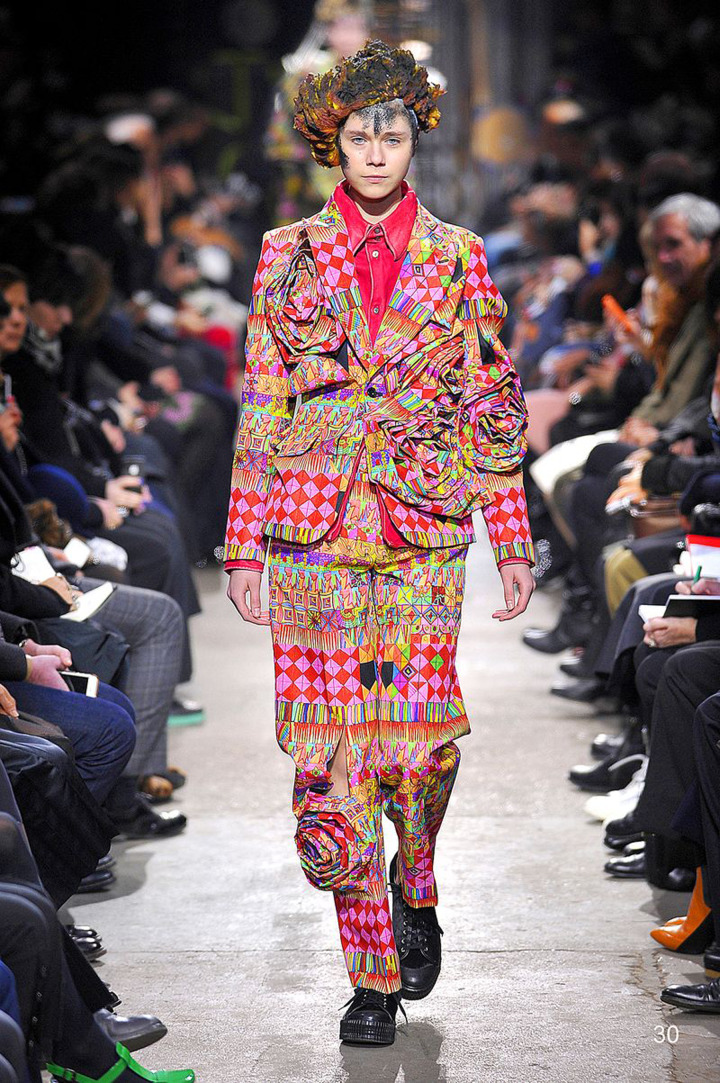
Comme Des Garçons women's autumn–winter 2013 collection, inspired by the work of Dan Michiels. Image 2 from: https://www.vogue.com/fashion-shows/fall-2013-ready-to-wear/comme-des-garcons.

**Fig. 3.**
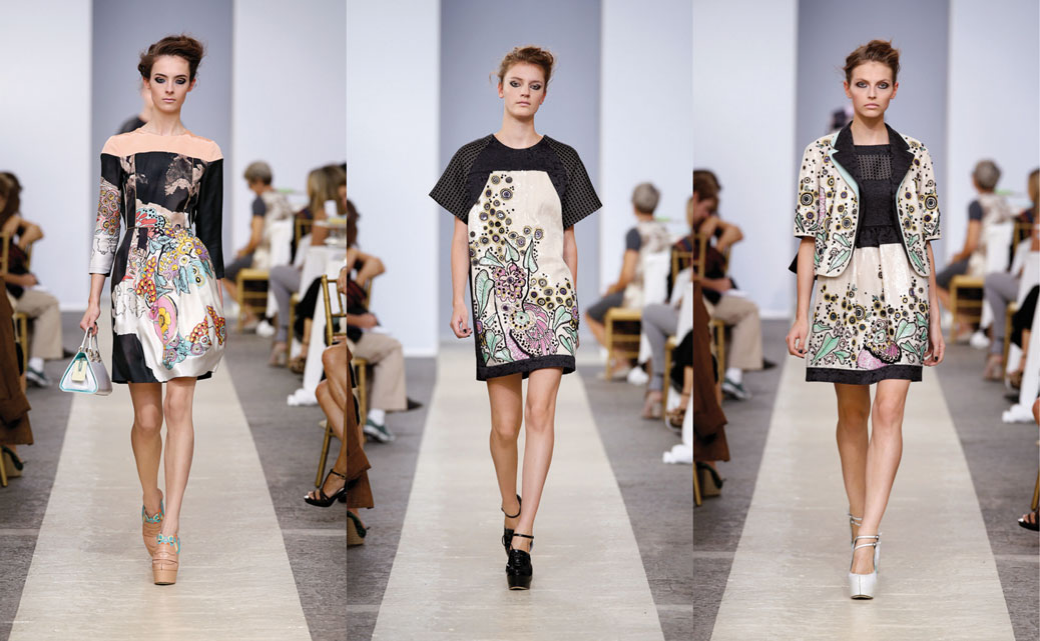
Antonio Marras, spring–summer collection 2013. Image 3 from: https://www.klatmagazine.com/interviews/antonio-marras-interview/8864.

**Fig. 4.**
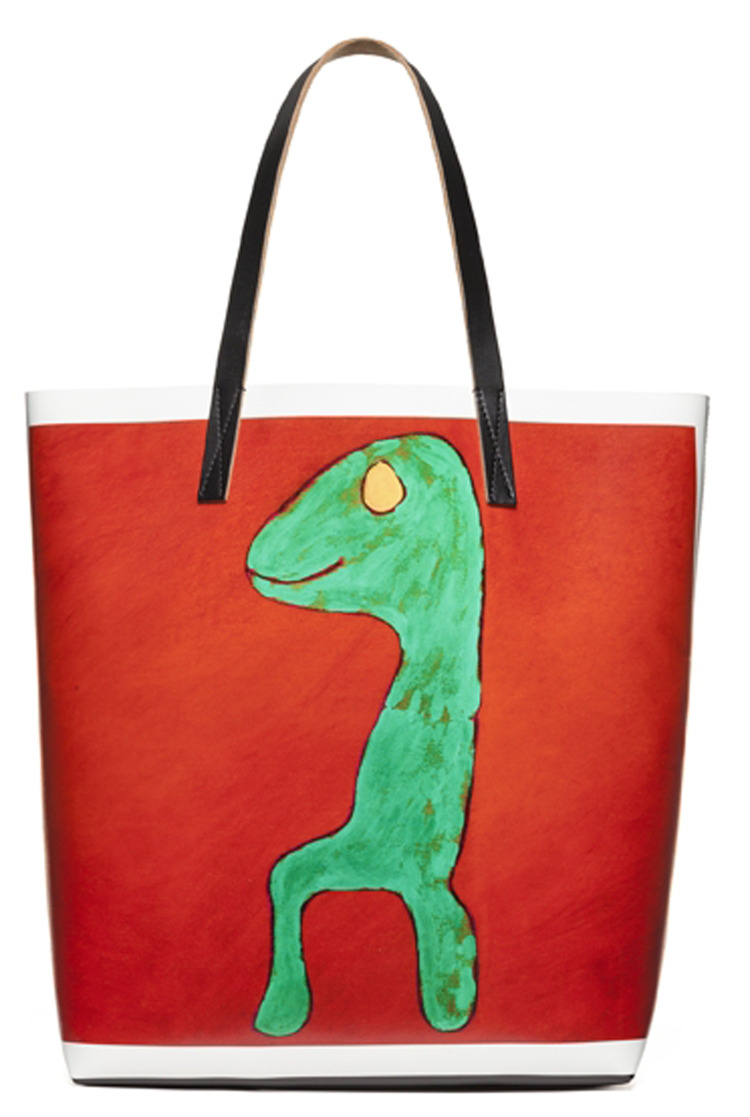
Hand bag, Marni pre fall 2014 collection, inspired by the work of Stefano Favaro. Image 4 from: https://www.vogue.it/galleries/gait13787

Antonio Marras's Vivian Girls were very different: in the spring/summer 2013 collection, Marras dedicated a tribute to Darger, after having studied the motifs and techniques of his work and having been deeply fascinated above all by its complex composition and sense of detail: he observed the layering created by the collage technique, which offered him the inspiration for the creation of details through applications of crystal embroidery. Visually, the clothes that most remind us of the original world of the Vivian Girls are those in which Marras has printed his own reworkings of Darger's work on fabric, the short dresses in pastel tones or the garments made with material layering. Marras then contextualised this fantasy world to the present day by proposing his own personal narrative for the Vivian Girls, imagining the fashion show setting as a real wedding.

In the Italian area another interesting experience involved the company Marni, founded and led by designer Consuelo Castiglioni, and French artists Christophe Joubert and François-Xavier Tavy-Sacley, and Italian Stefano Favaro. The three authors were contacted through the associations of which they are members (respectively Esat Menilmontant, Association Personimages both based in Paris and the Outsider art Observatory of Verona) to start a collaboration from which the pre/fall 2014 collection was born. Declared criteria include the formal freedom of the works, the spontaneity of the drawings and refined colour experiments. Stylised animal figures, floral motifs, abstract designs borrowed from the works were printed on short women's dresses, on long skirts, on both men's and women's shirts, but above all on T-shirts, backpacks, leather bags, clutches and handbags.

What emerges from this brief analysis of experiences is that the fashion world has always positioned itself as a space open to all kinds of interaction, looking to art as a source of creative inspiration, fascination or quotation. The fact that, in the last 15 years, designers and fashion houses have also started to draw on motifs found in outsider artworks is certainly no coincidence. The designers presented in these pages are known worldwide for their creative, innovative, eccentric and unconventional aesthetics, and when they work on the design of their collections, they naturally channel the cultural stimuli they experience into them. In the last 10 years outsider art has been much talked about, also thanks to the 2013 Venice Biennale Il Palazzo Enciclopedico, curated by Massimiliano Gioni, where works by self-taught artists were exhibited precisely in the perspective of dialogue and juxtaposition between ‘outsider and insider’. In any case, I believe that these interactions can contribute to making the works of outsider authors known and to promoting that process of contamination between different spheres that sees outsider art increasingly included in the dynamics of the contemporary art world.

## Appendix

For more information on creative ateliers:

https://creativegrowth.org/

https://www.musevery.com

https://lasgrandatelier.be/

https://www.creativityexplored.org/

http://www.esatmenilmontant.com/

https://www.personimages.org/

https://www.lao-art.it/lao/

## About the author

Giulia Pettinari is an Italian art curator and a state public school teacher. After her graduation in Visual Arts at the University of Bologna she began to dedicate herself to outsider art. Since 2020 she is deputy editor of the outsider art magazine Bric a Brac. She is co-founder of the cultural association McZee, based in Montecassiano (Macerata, Italy). Among her latest publications are: Intorno a un tavolo. Antonio Tolomei e altre storie del manicomio Santa Croce di Macerata, 2018; SIC12 artstudio: la collezione Giacosa – Ferraiuolo a Roma (2022).
